# Peculiarities of Enhancing Resistant Starch in Ruminants Using Chemical Methods: Opportunities and Challenges

**DOI:** 10.3390/nu5061970

**Published:** 2013-06-04

**Authors:** Kathrin Deckardt, Annabella Khol-Parisini, Qendrim Zebeli

**Affiliations:** Department for Farm Animals and Veterinary Public Health, Institute of Animal Nutrition and Functional Plant Compounds, University of Veterinary Medicine Vienna, Veterinaerplatz 1, Vienna 1210, Austria; E-Mails: kathrin.deckardt@vetmeduni.ac.at (K.D.); annabella.khol-parisini@vetmeduni.ac.at (A.K.-P.)

**Keywords:** resistant starch, ruminally resistant starch, ruminant health, barley, chemical grain processing

## Abstract

High-producing ruminants are fed high amounts of cereal grains, at the expense of dietary fiber, to meet their high energy demands. Grains consist mainly of starch, which is easily degraded in the rumen by microbial glycosidases, providing energy for rapid growth of rumen microbes and short-chain fatty acids (SCFA) as the main energy source for the host. Yet, low dietary fiber contents and the rapid accumulation of SCFA lead to rumen disorders in cattle. The chemical processing of grains has become increasingly important to confer their starch resistances against rumen microbial glycosidases, hence generating ruminally resistant starch (RRS). In ruminants, unlike monogastric species, the strategy of enhancing resistant starch is useful, not only in lowering the amount of carbohydrate substrates available for digestion in the upper gut sections, but also in enhancing the net hepatic glucose supply, which can be utilized by the host more efficiently than the hepatic gluconeogenesis of SCFA. The use of chemical methods to enhance the RRS of grains and the feeding of RRS face challenges in the practice; therefore, the present article attempts to summarize the most important achievements in the chemical processing methods used to generate RRS, and review advantages and challenges of feeding RRS to ruminants.

## 1. Introduction

Proper feeding of ruminants is important to maintain a high health status of the animal, and also critical to ensure that milk and meat are produced in an efficient and cost-effective manner [1]. In high-producing ruminants, such as dairy cows or feedlot cattle, the energy requirements are high to support high milk yields and rapid weight gains. Therefore, these intensive management systems typically encourage the inclusion of large amounts of easily degradable carbohydrates in the diet to support a high performance and enhance cost efficiency [2]. Besides providing energy for the host, grains in the diet also present the most essential energy source for the rumen microorganisms, because glucose is needed for bacterial growth and, hence, for microbial protein synthesis [3]. However, although these feeding practices are useful to maximize the production in a cost-effective manner, they do not cope with the digestive physiology of cattle. Large amounts of cereal grains in the diet are fed at the expense of the proportion of structural dietary fiber, which is needed to maintain rumen health [4].

The most common cereal grains used in ruminant nutrition are barley, maize, and wheat. In contrast to maize, barley grain is rich in rapidly fermentable starch, resulting in a more rapid accumulation of short chain fatty acids (SCFA) in the rumen fluid [2,5]. For instance, depending on the amount of dry matter ingested, the rumen of dairy cows may generate up to 120–130 mol (6–7 kg) SCFA daily, in which is almost 70% of the energy is supplied to the host [5]. This load of SCFA leads to acidotic conditions in the rumen, commonly known as subacute ruminal acidosis (SARA) [6]. If the ruminal pH drops as low as around pH 5, this eventually results in an acute ruminal acidosis (ARA) [7,8]. ARA and SARA are severe metabolic diseases in cattle associated with impaired digestion [9,10], frothy bloat [11], laminitis [2,12], liver abscesses [13], and polioencephalomalacia [14] in cattle. Moreover, the aforementioned diseases are linked to a sub-optimal performance, and a reduced welfare, of the animals, consequently leading to a significant impact on the profitability of beef and dairy industries.

The development of effective feeding strategies for ruminants requires the maintenance of an optimal rumen metabolism. In this regard, because the amount of fiber in the diet of high-producing ruminants is limited, slowing down the rate of ruminal degradation of starch-rich cereals would reduce the starch availability for microbial degradation in the rumen and, therefore, minimize the risk of SARA and ARA [15]. Indeed, feeding cereals rich in starch that resists rumen fermentation has been suggested as an alternative strategy to help avoiding rumen disorders [16]. In ruminants, starch that resists microbial enzymatic degradation in the upper gut sections (*i.e.*, forestomachs) may be termed as bypass starch, undegradable starch, or ruminally resistant starch (RRS). More precisely, RRS is expected to resist degradation mechanisms in the rumen and be digested in the small intestine. For simplicity this term is used throughout this review. During the last two decades, a large number of studies have examined ways to modulate the rumen degradability of typical cereal grains, aiming to improve the feed efficiency of cattle by altering the nature and amount of the starch available to rumen microbiota, and hence shifting some starch digestion to the small intestine [17,18,19,20,21].

A substantial body of evidence and several review articles exist about the use of mechanical-thermal processing methods of grain [17,18,19,20,22]. The advantages and disadvantages of several “old” chemical methods that use chemicals like NaOH, aldehyde, and ammonia have also been summarized recently [21]. However, their relevance with regards to the modulation of starch degradation and the metabolic effects of feeding RRS in ruminants are not discussed. In addition, during the last few years, the use of some new chemical methods that utilize weak organic acids [23,24,25] or tannic acid [26] to confer the starch resistance against amylolytic degradation has been proposed, but there is no report summarizing the available data. The present article attempts to summarize the most important achievements in the chemical processing methods used to generate RRS, and review the advantages and challenges of feeding RRS to ruminants, including its effects on rumen health and metabolism.

### 1.1. Grain Histological Features

The structural elements of grains are the pericarp (bran) serving as a barrier and protecting the inner germ (embryo) and endosperm. The proportions of the different components vary among grains [27]. The main function of the pericarp and the germ is water intake regulation. There is only a small starch content in these two structures. The endosperm contains the major part of starch [28] and consists of four layers, starting with the outer aleurone layer, followed by the subaleurone layer (peripheral endosperm), corneous endosperm, and the inner floury endosperm. The aleurone layer contains important enzymes as well as enzyme inhibitors, vitamins, and minerals [29], whereas the peripheral and corneous endosperm embody starch granules and are enclosed by an impenetrable protein-matrix. The innermost layer, the floury endosperm, contains most of the starch granules and is very sensitive to enzymatic reactions and grain processing because there is no protein-matrix attached. The fractions of the peripheral, corneous, and floury integuments differ between grain species. Due to the diversification of the endosperm layers, grains are called waxy, non-waxy, vitreous, opaque, and flinty. Waxy species, which contain more amylopectin than amylose (see chapter *1.2.*), are digested faster than non-waxy species *in vitro* [30,31]. Further, the distribution of amylose is different between barley cultivars. In waxy barley, the amylose content is higher in the subaleurone layer than in the inner parts of the endosperm compared to a non-waxy cultivar. However, amylopectin distribution was similar in those two cultivars [32].

### 1.2. Is Starch Always Starch?

The main component of most cereal grains is starch, accounting for 70% to 80% of the dry matter (DM) content [3]. In a recent review paper of Zebeli *et al*. [4] starch contents of maize grain (70.3% of DM), wheat grain (67.6% of DM), and barley grain (57.8% of DM) were presented. According to Huntington *et al.* [33], wheat contained the major proportion of starch (77% of DM), followed by maize (72% of DM), and barley (57%–58% of DM). It has to be considered that geographical, genetic, and environmental factors as well as agricultural methods and experience are responsible for the observed variation of the chemical compositions of cereal grains [21,30].

**Figure 1:**
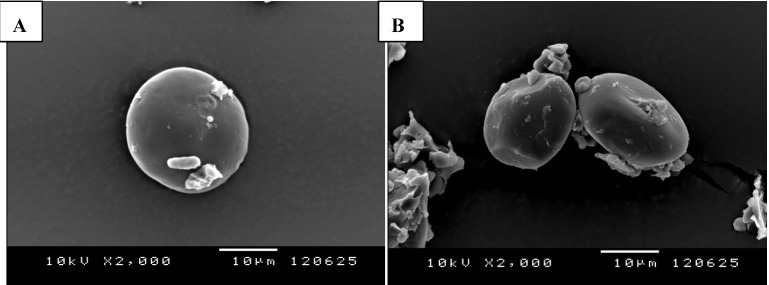
Scanning electron microscope images of starch granules of “Eureka” a 2-row-winter-feed barley cultivar (**A**) and “Vienna” a 2-row-summer-feed barley cultivar (**B**) (2000×).

Starch, a polysaccharide molecule consisting of α-d-glucose units, is the most important nutrient reserve of plants. Most starch is located in the floury endosperm, consisting of compact granules (reviewed by Pérez and Bertoft [34]). Starch granules vary in magnitude from 1 to 100 μm [35,36] and differ between grain types. According to scanning electron microscope images, the size of starch granules is comparable between barley cultivars (Figure 1). Environmental factors (e.g., different temperature, location, rain fall) may influence the size of the starch granules and their characteristics [37]. Lipids, such as free fatty acids and phospholipids, as well as proteins, are associated with starch granules and can play an important role influencing their digestion [20,38]. The major components of starch granules are amylose and amylopectin. Starch usually consists of 20%–30% of amylose with α-d-(1-4) linkages. A certain grain type called amylomaize contains about 65%–70% [20], or 40%, [39] of amylose. In contrast, amylopectin consists of α-d-(1–4) linkages branched with α-d-(1–6) linkages after 20 to 25 glucose units. These molecules are connected by hydrogen bondings [3,34]. Amylose chains can either form single or double helices, have a molecular mass of about 1 × 10^5^ g/mol [40], and a degree of polymerization (DP) up to 4400 [41], 6000 [42], or 6680 [43]. The two known types of amylose, type A and type B, differ only slightly in terms of H_2_O units per cell and arrangement of their double helices. However, amylopectin is one of the most colossal molecules on earth, with 5 × 10^6^−5 × 10^7^ g/mol [40], and an average DP of two million [42]. Due to the intense branching of amylopectin, cluster models were created for illustrating its individuality [42]. The contents of amylose and amylopectin can be different between cultivars. Waxy grains, for example, are composed of amylopectin alone and have the ability of accelerated swelling in heated water, as well as more rapid *in vitro* and *in vivo* digestion in comparison to non-waxy genotypes [30]. In addition, starch granules are described to be semicrystalline [35], because they contain both, water impermeable crystalline structures (about 30%, mainly consisting of amylopectin) and hydro pervious amorphous regions (about 70%, primarily embodying amylose; [42,44]).

There are three types of native starch, including type A, B, and C, which differ in amylopectin chains lengths and compactness, as well as water attendance in the starch granules. Type A is most common in cereals, whereas type B occurs predominantly in raw potatoes and banana, and type C is characteristic for beans and peas. Starch types A–C were detected by means of X-ray diffraction. Types A and B posses real crystalline structures, whereas type C is a mixture of type A and B (reviewed by Sajilata *et al*. [42]). Another classification of starch was established with regard to enzymatic interactions, such as rapidly digestible starch (RDS), slowly digestible starch (SDS), and resistant starch (RS). Rapidly digestible starch and SDS are completely digested by the intestinal enzymes of humans and animals. In contrast, RS cannot be degraded by α-amylase in the duodenum of monogastrics; therefore, RS is degraded in the large intestine [45,46].

In ruminant nutrition, starch that is able to withstand ruminal degradation mechanisms (e.g., amylolytic bacteria and protozoa), the RRS, is mainly digested to glucose and absorbed in the small intestine [24]. In monogastrics, RS was first mentioned by Englyst *et al*. [47] and there are several more recent studies on resistant starch acting as prebiotic in human nutrition [48,49,50,51]. There is also evidence that RS has the potential to reduce human colon cancer and colitis risks [52,53], as well as type 2 diabetes risks [54,55].

Four types of RS (e.g., RS_1–4_ or RS type 1–4) have been described so far [42,56,57,58,59]. Type 1 RS is heat stable and digestive enzymes have no access due to its physical characteristics. Partly or whole milled cereal grains contain RS_1_. Resistant starch 2 (RS_2_) possesses an ungelatinized (very compact) granular structure, and is resistant to digestive enzymatic processing. Sources containing RS_2_ are raw potatoes, green bananas, and high amylose maize. The most common RS in food is RS_3_, a retrograded starch, derived from cooled gelatinized starch that is totally inaccessible by amylases. Bread, cooked and cooled potatoes, and maize flakes are numbered among RS_3_-sources. Chemically rearranged starches (unnatural starches), which have other chemical bonds than α-d-(1–4) and α-d-(1–6) linkages belong to the RS_4_ type. Grains processed with chemicals, e.g., acetylated indica rice [60] or maize grain, modified with sodium sulfate/trimetaphosphate/tripolyphosphate [61], typically contain type 4 RS. In addition, there are various factors, such as certain characteristics of starch (e.g., crystallinity, granular structure, amylose:amylopectin ratio, retrogradation of amylose, amylose chain length), which have a great influence on RS formation (reviewed by Sajilata *et al.* [42]). It is clear that starch is a very ambivalent molecule, both chemically and from the degradation point of view. There seem to be differences in describing starch both as a native starch within a certain grain, as well as between various processing methods of grains, which considerably differ in their potential to generate RS. The latter will be treated in more detail in the sections below.

## 2. Enhancing RRS Starch Content in Concentrates for Ruminants

The amount of native resistant starch found in grains can vary considerably. For barley, for example, which is an important grain used in the feeding of ruminants, about 6% of 209 tested spring varieties were found to have a very high RS content (11%), whereas 7% showed a very low RS content of <1% [62]. As early as in the mid-1960s, cereal-processing technologies with barley, maize, and sorghum were developed [63]. Mechanical or thermal treatments, such as steam flaking, roasting, popping, reconstituting, or micronizing, were performed in order to enhance the starch digestibility in the rumen and to increase the feed utilization efficiency [17]. However, the negative effects of feeding diets rich in degradable starch, such as high incidences of ARA and SARA, which were observed in the intensive rearing systems of ruminants from the 1980s onwards, changed this trend of grain processing in cattle (reviewed by Owens *et al.* [7]). For this reason, many attempts have been made to develop grain processing technologies to promote the animals’ performance and feed utilization, but without impairing animal health. Physical and thermal treatments of grain, in relation to performance in cattle, have been reviewed more often [17,21] than the chemical processing techniques.

2.1. “Traditional” Chemical Grain Processing Methods: Do They Enhance RRS and Cattle Health?

Chemical grain processing methods employ various chemical substances aiming to modulate the starch structure and, hence, their degradation characteristics. Compared with mechanical-thermal processing techniques, the chemical methods have advantages because they are cheaper, which often is a precondition for a technique to be applied in practice. The advantages of treating grain with chemical substances were observed with the use of sodium hydroxide (NaOH), which resulted in a slower ruminal starch degradation as well as a decreased susceptibility to rumen acidosis [64]. Treating barley grain with 3%–4% NaOH also increased the whole tract digestibility (reviewed by Dehghan-Banadaky *et al.* [21]). However, sorghum treated with NaOH showed a reduced total starch apparent digestibility when measured in the entire gastrointestinal tract [65]. A decreased starch digestibility means that the sorghum starch treated with NaOH was recovered in the feces, and this is not desired because low digestibility impairs feed efficiency in cattle. These results indicate that the type of grain itself also has to be taken into consideration when processing grain with NaOH. Unfortunately, the use of NaOH also has negative side effects, which preclude the use of this technique as a routine method in the practice. These side effects include the fact that prolonged feeding of high amounts of NaOH can lead to nephrotoxicosis in cows [66] and soil salinification [21], whereby the handling of this corrosive chemical can be seriously hazardous for the users. In addition, the nutritional quality of barley was decreased by its treatment with NaOH, with the contents of essential amino acids lysine and cystine declining [67] and the quantity of vitamin E being below the limit of detection [68]. The mode of action of NaOH is described in [69]. It is explained that NaOH treatment caused some physicochemical changes, such as swelling of the starch granules, and amylose and amylopectin leaching out of the structure. The released amylose and amylopectin molecules formed a continuous gelled matrix, which was surrounding the residues of the ruptured starch granules. Due to these findings, it can be said that NaOH treatment had an effect on the rheological properties of starch.

Besides NaOH, formaldehyde (HCHO) is another chemical that has been widely used to treat grains. In a study by Martínez *et al*. [70], 40 goats were fed a wheat-based diet protected with 5% HCHO, and mixed with 15% saponified tallow. Compared to the control group, the numbers of follicles were enhanced in the goats fed the formaldehyde-treated wheat, indicating a better energy supply and metabolic health status of the animals fed the latter diet. Indeed, the authors concluded that the follicle development was stimulated by RRS reaching the duodenum and the subsequent glucose supply, which was apparently increased by this chemical treatment. An increased glucose supply enhances the insulin level, influencing the gonadotropin secretion or the follicles directly [71]. However, the positive effects of feeding RRS on insulin and ovarian function were not supported by the results of Garnsworthy *et al.* [72], who fed cows RRS of flint maize (not chemically modified), and reported no influence on plasma insulin and ovarian function, compared to the controls. Fluharty and Loerch [73] conducted three experiments (two *in vitro*, and one *in vivo* trials) concerning formaldehyde (37% wt/wt HCHO) treated maize grain. In the first *in vitro* study, maize treated with 1%, 2%, and 3% HCHO, milled through a 1 mm screen, reduced the *in vitro* dry matter disappearance while increasing the pH value with rising concentrations of HCHO, compared to the 0% HCHO treated maize. In the second *in vitro* experiment, maize was treated with 0, 0.5%, and 1% HCHO, and ground to pass a 1.9 cm sieve in order to be close to *in vivo* conditions. The 0.5% and 1% HCHO treatment reduced the *in vitro* dry matter disappearance and increased the pH value as well, possibly due to the lower amount of SCFA produced. In a following *in vivo* trial, with six abomasally fistulated wethers 1% and 2% HCHO-treated maize (ground to 0.95 cm), indeed, withstood the degradation mechanisms in the rumen. Ruminal starch degradation significantly declined with rising HCHO concentrations while total tract starch digestion was not negatively affected, which indicates that the RRS was degraded in the small and large intestine, compensating for the lower rumen degradation of RRS. Similar results were shown in the study by Oke *et al.* [74], who reported an enhanced flow of starch from the rumen to the small intestine due to an HCHO treatment of maize. Nearly 48% of the treated maize and only 24% of the untreated maize reached the small intestine, and the ruminal starch degradation was reduced by 38%, compared to a native maize diet.

In another experiment HCHO treated barley grain was used in an *in vitro* gas production test to detect DM fermentation *in vitro* [75]. The results showed that treated barley significantly reduced the gas volume (ranging from 33.3 to 51.0 mL/g organic matter for each barley cultivar) and influenced the gas production kinetics *in vitro*. However, since this study was only conducted *in vitro*, no conclusion can be drawn about the treatment’s influence on animal performance. In a study by Ortega-Cerrilla *et al.* [76], sheep and cows fitted with ruminal and duodenal cannula were fed with 30 g HCHO/kg barley crude protein (CP). Rumen degradability of DM, starch, and total N, as well as the flow of nutrients to the small intestine were determined. Although the barley treatment caused a significant resistance to rumen degradation processes, compared to untreated barley, there was no increased flow of starch to the duodenum. Although HCHO-treated grain showed some positive results concerning the site of starch digestion and enhancing the amount of RRS, environmental and health issues have to be considered thoroughly when using HCHO as a chemical to treat feedstuffs for animal nutrition.

The ammoniation of grain was tested in the past with some experiments conducted in cattle. Cows were fed with barley grain soaked with different concentrations (6.5, 13, and 19.5 g/kg barley) of ammonia in an *in situ* experiment [77]. Interestingly, the animals tended to eat the feed more rapidly with increasing ammonia concentration, but this feeding habit did not increase overall DM intake. Unfortunately, the mean pH value declined and the time of the rumen pH below pH 6 was prolonged with increasing concentrations of ammonia, which disturbed the rumen physiology. However, ammonia-treated barley reduced the *in situ* DM and starch degradation, thus increasing the RRS content. In another *in vivo* experiment from the same team, ammonia treated barley did not affect DM and starch intake, or digestibility [78]. Milk yield increased with the rising concentration of ammonia. Moreover, the trial supported the previous results by slowing down the ruminal degradation of starch from processed grain.

Despite the advantages provided, the use of ammonia, aldehyde, and NaOH treatments are very laborious and intensive methods because the grain has to be soaked for a long period of time [21]. In addition, along with health consequences when applied during long periods of time, some of the traditional methods mentioned above are corrosive, pose health risks to laborers and possibly even consumers, and require special equipment. In order that a processing technology be adopted by the dairy industry it must be cost-effective, provide outcomes that justify its use, and be applied easily under farm conditions [24]. As mentioned above, the results of chemical processing of grain depend on the type of chemicals used, their concentrations, as well as the type of grain treated itself. Thus, it seems that a substantial research work is still warranted to identify chemical substances and their concentrations with good results in lowering starch degradation without impairing animal health and the health of the laborers.

### 2.2. New Chemical Grain Processing Methods and Potential Metabolic Effects in Cattle

There is an up and coming interest in detecting new chemical grain processing techniques, such as treating grain with mild acids in order to modify starch degradation. Only a few experiments were conducted under *in situ* and *in vivo* conditions in ruminant nutrition so far [24,25,26,79,80], hence there is scarcity of information and further studies are warranted.

Lactic acid bacteria, and their metabolized product lactic acid (LA), have been used for fermentation and preservation of food for centuries, in dairy- [81] or non dairy-fermented products [82]. However, only recently has research indicated interest to use LA as a modifier of the cereal grain starch. For example, bread, using dough with LA, has been shown to be an efficacious nutritional therapy to improve gluten-free bread for celiac disease patients [83]. Interestingly, the food industry has also successfully used LA to modify starch degradation features. Lactic acid has the ability to slow the enzymatic action of amylases of grain, which led to a decrease in degradability of starch in human and *in vitro* studies [23,84]. The exact mode of action of LA on starch structure is currently not fully understood. One possibility could be that LA causes a linearization of the branched amylopectin-molecule and hence limits enzymatic attack [85,86]. Another explanation could be that interactions between gluten and LA can provide a barrier for enzymatic degradation [87].

In another study by Östman *et al.* [88], ten healthy men and women consumed bread with or without LA-treated dough for breakfast. After four hours they all ate a high glycaemic index lunch (e.g., commercially available fried meatballs, mashed potatoes, canned sweet maize). Before and after the meals, blood glucose, as well as insulin levels, were measured. Both the glucose levels and the insulin response were significantly reduced in the LA group, 30 to 45 min after the lunch meal. The authors concluded that bread prepared with LA-treated dough potentially improves the second meal glycaemia. In a further study by Östman *et al.* [89], feeding bread containing LA to obese and hyperinsulinemic rats ameliorated their glucose intolerance, possibly due to the effects of LA on the reduction of the degradation rate of starch in the gastrointestinal tract of animals.

However, only very few such experiments have been conducted in livestock so far [24,25,79,80]. In those experiments [24,25], barley grain steeped in 0.5% or 1% LA was tested in late-lactating dairy cows to evaluate the effects on the rumen fermentation profile, immune status and productivity, as well as decreasing the risk of rumen acidosis. Although barley is rich in energy and protein, and an excellent feed grain for ruminants, feeding barley grain often leads to digestive and metabolic disorders, often in connection with a high incidence of SARA [6,15]. This is due to the rapid fermentation rate of barley starch. Between 80% and 90% of barley starch, but only 55% to 70% of maize starch, are degraded in the rumen [4,90]. Interestingly, the study by Iqbal *et al.* [24] showed that unlike the control group, the ruminal pH value of the LA-treatment group was above SARA values (ruminal pH 5.8; Zebeli *et al.* [15]), indicating a slower degradation of barley starch in the rumen because of the treatment with LA. The pH value in this study was measured 10 to 12 h after feeding, which is the most intensive time of fermentation. The concentration of SCFA in the rumen fluid also declined due to the increase of RRS in the diet in response to the LA-treatment of barley, which explained the higher rumen pH. Furthermore, several acute-phase proteins, such as haptoglobin (Hp) and serum-amyloid A (SAA) were decreased in the plasma of cows fed the LA-treated barley, which was interpreted as improvement of the immune status of the animals. Most interestingly, the concentrations of glucose, insulin, and cholesterol, all variables indicating the energy status, were higher in the treatment group [25], indicating that the increase of RRS in the diet by LA treatment was reflected in more net glucose being transferred from the small intestine to the liver. The greatest difference in the effects of feeding resistant starch in ruminants and monogastrics is that in monogastrics the RS will be degraded to SCFA in the large intestine, while in ruminants, the main proportion of RRS will be degraded to glucose in the small intestine (see the section below for more details in the underlying mechanisms of this process).

In a subsequent study from the same team [79], barley grain was treated with 1% LA and was heated in the oven at 55 °C for 48 h because heat treatment had already revealed positive results in a former *in situ* study concerning starch degradation [91]. Again, the ruminal pH value of the cows fed treated barley was higher than that of the control group. A decrease and a change in the profile of SCFA were observed in the animals fed the treated barley grain. The concentrations of acetate and butyrate increased, while propionate decreased. High values of acetate were positively correlated with an elevated milk fat content, which could be confirmed in this experiment. In these crossover studies, using eight cows in each study, treating grain with LA showed the most promising effects concerning the starch degradation in the rumen, thus providing optimal conditions for healthy and productive animals. However, more research with large cohorts of animals and under practical conditions is needed before this method may be introduced in practice.

In fact, organic acids are naturally found in biological tissues or produced in the gastrointestinal tract, and they are generally used to modify rumen fermentation. Among them, fumaric, malic, and aspartic acids were, so far, the most frequently used acids in ruminant nutrition [92,93]. Fumarate and malate are intermediate products of the citric acid cycle, as well as intermediates in the succinate-propionate pathway of *Selenomonas ruminantium*, predominant in the rumen ecosystem and known to stimulate proprionate production and increase pH value, because of its potential to increase the uptake and utilization of lactic acid. The most promising additive is malate, which has several benefits, such as increasing DM digestibility, decreasing methanogenesis, and uncomplicated application (intensively reviewed by Khampa and Wanapat [94], and Castillo *et al.* [95]. However, due to the high costs of malic acid, this feed additive is not the best choice with regard to the farmer’s budget [93]. Unfortunately, none of the above mentioned experiments evaluated the potential of organic acids in terms of slowing down ruminal starch degradation.

Tannins, naturally occurring secondary plant constituents, are also suggested as a means to slow down ruminal starch degradation [26]. Barley grain was milled to pass a 2 mm sieve and was subsequently soaked in 0 (served as control), 1%, 2.5%, and 5% of tannic acid (TA) for 20 min. Then, the treated barley was dried in a forced-air oven at 45 °C and was incubated in the rumen of four ruminally fistulated ewes using an *in situ* method. Measurements of the disappearances of DM and CP at 2, 4, 8, 12, 24, and 48 h indicated a reduced rumen DM and CP degradability with rising concentrations of TA. Tannic acid, known to bind to protein and fiber components [96,97], may also form complexes with starch and therefore could be a promising tool for protecting starch from ruminal degradation. Moreover, it is well known that tannins are toxic to some species of rumen microbiota and change their composition, which could impair rumen degradation [97]. However, since only limited data exist about the potential role of TA on rumen degradation of barley, and these data are from *in situ* studies only [26], intensive *in vivo* research is warranted to validate these *in situ* data before conclusions for practical use can be drawn.

### 2.3. The RRS in Ruminants Lowers the Risk of Acidosis and may also Provide Energy—A Model

The fate of degradable non-resistant starch (NRS) in the rumen of cattle is degradation either by protozoa that engulf starch granules or amylolytic bacteria that secrete α-amylases, which break down α-d-(1–4) linkages. Maltose, maltotriose, and small amounts of free glucose are the end products of amylose- as well as of amylopectin-debranching, including the degradation to α-limit-dextrins [98]. These oligosaccharides, still consisting of α-d-(1–6) linkages, are finally degraded by other enzymes, such as R-enzyme, pullulanase, iso-amylase, or α-limit dextrinase [98]. Glucose molecules are subsequently fermented in bacterial cells via the Embden-Meyerhof-pathway (glycolysis) and pentose-phosphate-cycle. Two molecules of pyruvate, as well as energy in the form of ATP and two molecules of NADH, are generated in a ten-step-cycle out of one glucose molecule [99]. The final products of microbial starch degradation are SCFA, mainly acetate, propionate, and butyrate, but also lactate, valerate, caproate, and iso-valerate are produced from pyruvate. The major SCFA are acetate, propionate, and butyrate with molar proportions in the ruminal fluid ranging between 45% and 70%, 15% and 40%, and 5% and 20%, respectively [100]. Diets rich in grain (>50% in DM) promote higher proportions of propionate at the expense of acetate, whereas butyrate is mostly unaffected by the grain:forage ratio in the diet. The metabolic processes of conversion of glucose to pyruvate and to SCFA are very rapid in the rumen. This is the reason why glucose and pyruvate can only be found in low amounts in rumen fluid [101]. Lactate is accumulated in high amounts only during ARA, whereas during SARA lactate is converted to propionate and also butyrate by lactate-utilizing bacteria in the rumen [101].

**Figure 2:**
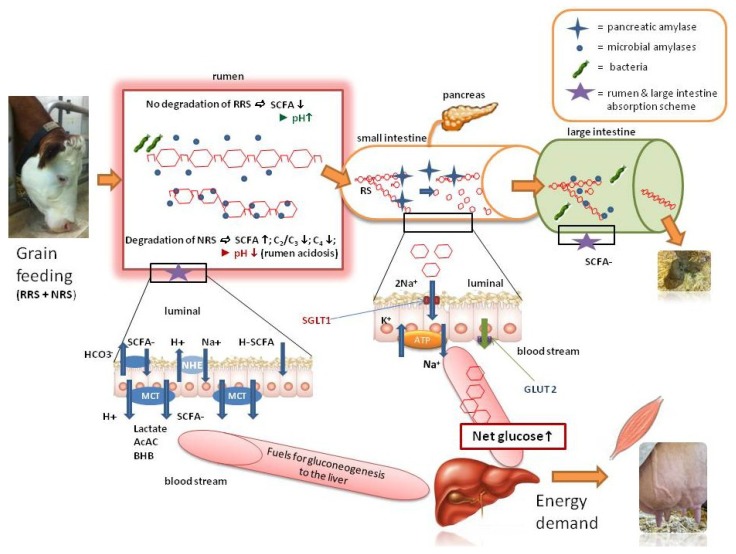
A simplified model describing the fates of ruminally resistant starch (RRS) and non-RRS (NRS) fed to cattle. The RRS is not digested in the rumen, which results in low concentrations of short-chain fatty acids (SCFA) and a higher ruminal pH. NRS is degraded in the rumen and leads to a release of SCFA, changing the proportions of acetate (C_2_): propionate (C_3_) and butyrate (C_4_), as well as decreasing the ruminal pH (high risk of rumen acidosis). The undigested RRS is mostly degraded in the small intestine by pancreatic amylases, some portions of it can be degraded in the large intestine. The model also indicates the mechanisms of removal of SCFA (metabolism of SCFA to beta-hydroxybutyrate (BHB), acetoacetate (AcAc), and lactate) from the rumen and the absorption of glucose from the small intestine.

Figure 2 shows the fate of dietary NRS in cattle, in a simplified scheme. The produced SCFA are carried into the epithelial cell as dissociated molecules, using the anionic exchange of HCO_3_^−^ or via non-ionic diffusion. In particular, the reticuloruminal absorption of SCFA has a dual, and thereby central, role in digestive efficiency and health of ruminants [5]. The absorption of SCFA ensures the direct recovery of energy substrates from the rumen into the metabolic pool of the animal. On the other hand, the absorption also regulates the intraruminal milieu and pH by the extraction of protons together with SCFA, hence lowering the risk of rumen fermentation disorders [102]. In order for dairy cows to avoid accumulation of SCFA and reduce the risk of ruminal acidosis, the decrease of the amount of NRS in the diets of dairy cows was reported as an efficient alternative [3]. However, more recent modeling approaches [15] revealed that about 15% of NRS from grains (*i.e.*, 3.8 kg NRS from grains per day for cows consuming about 25 kg DM/day) may be considered as a general optimum to assure normal rumen conditions and digestion. In the rumen epithelial cells, SCFA are either metabolized intraepithelially to ketone bodies (most importantly to β-hydroxybutyrate) and lactate, or directly transferred into the bloodstream by means of MCT (monocarboxylate transporters;) [103]. A range of MCT such as MCT 1, MCT 4, and MCT 2 have been identified at the mRNA level in the rumen epithelium [104]. Finally, the metabolized products of SCFA or the original SCFA arrive at the liver via portal circulation, where ketone bodies serve as fuels and lactate and propionate are utilized for gluconeogenesis ([99,105], see Figure 2).

The best way to increase the glucose supply for ruminants is to include high levels of RRS in the diet to ensure enough glucose is reaching the liver [30]. In fact, the energy from direct glucose supply can be used more efficiently by the animal because the ruminal anaerobe glycolysis of starch glucose, and the subsequent hepatic gluconeogenesis of propionate both show a low energy efficiency. The simplified model of Figure 2 describes the fate of RRS in ruminants. In contrast to NRS, RRS is not degraded by rumen bacteria, but directly passed on to the small intestine, were pancreatic α-amylases degrade most of the starch matrix, except for RS that might resist duodenal enzymatic digestion and flows to the large intestine. In the small intestine, α-amylases work similarly to bacterial amylases, which degrade starch into oligosaccharides and dextrines. At the brushborder membrane of the duodenal epithelial cells, maltase-glucoamylase, trehalase, and lactase deconstruct oligosaccharides into glucose molecules. Interestingly, the isomaltase-sucrase level in the ruminant is not detectable compared to monogastrics [106]. After that, glucose is connected to a sodium-glucose transporter (SGLT1) in exchange with two molecules of sodium and enters the epithelial cell. By means of a GLUT 2 (basolateral glucose transporter) glucose is carried into the interstitium where it can enter the bloodstream. The main starch digestion is performed in the proximal third of the duodenum [30,106]. However, Huntington *et al.* [33] question the use of SGLT1 by glucose molecules, because of its lack of adaption to the quantity of glucose molecules supplied. Glucose molecules can also enter the epithelial cell by solvent drag (paracellular transport). This mechanism is only possible at pathological conditions, when glucose reaches very high concentrations in the lumen (>25 mM) [106].

There are concerns about a restricted capacity of starch digestion in the small intestine of ruminants, due to the limited availability of pancreatic α-amylases and low glucose absorption (reviewed by Huntington [30,33]; Matthe *et al.* [16]; Cerrilla and Martínez [98]). Matthe *et al*. [18] recommended not to include more than 1.3–1.8 kg RRS per cow per day in order to avoid a decrease of starch utilization efficiency, although a good adaptation of the activity of digestive enzymes and an increased glucose supply resulting in an increased milk yield are reported after the supply of RRS in cattle (reviewed by Cerrilla and Martínez and Reynolds [98,107]). The aforementioned experiments of Iqbal *et al.* [24,25,79] also confirmed positive effects on the metabolic health status by potentially shifting starch degradation from the rumen to the intestine (higher glucose, insulin, and cholesterol in the plasma), but losses of microbial protein in the excrements and a decreased milk fat content were also reported [107].

As illustrated in Figure 2, starch that is not digested in the rumen or small intestine is fermented by microbiota in the large intestine. Nevertheless, hindgut starch fermentation is not desirable, in particular in terms of microbial protein losses [33], whereas the SCFA are absorbed across hindgut epithelia at similar rates as across the rumen epithelia [108]. The main health disadvantage of shifting starch fermentation to the hindgut is the hindgut acidosis, which occurs if too much starch reaches the large intestine, and the production of SCFA outweighs the absorption processes. Clinical symptoms are similar to rumen acidosis and the consequences might be laminitis, systemic inflammation, and poor health [108]. Thus, to avoid disorders in the large intestine of ruminants, the feeding of RRS must ensure that it is digested in the small intestine and only very low amounts can flow to the large intestine to be fermented there. Altogether, the best way to increase glucose supply in ruminants seems to include an optimal amount of RRS in the diet, thus enhancing the portal glucose supply, combined with a certain but limited level of NRS, for enough propionate to be converted into glucose in the liver without adversely affecting the fibre digestibility and other rumen functions.

## 3. Conclusions

Processing of grain to enhance the amounts of RRS in ruminants, or RS in monogastric species, is becoming increasingly important because this type of starch has health-enhancing properties. In ruminants, unlike monogastrics, the strategy of enhancing RRS is helpful, not only in lowering the risk of metabolic disorders and promoting digestion, but also in enhancing the net glucose supply for the host. Thus, enhancing the amount of RRS in the diets of ruminants not only alleviates the deficiency of dietary fiber, but also increases the energy efficiency by enhancing the net supply of glucose, which can be directly utilized by the host for growth or milk production. Despite the progresses made in using various processing methods to modify starch, more research is needed to improve these methods used to increase the amount of RRS in ruminant nutrition. The majority of the methods used so far have disadvantages due to the expensive, dangerous, polluting, or user-unfriendly chemicals used, such as sodium hydroxide or formaldehyde. The use of weak organic acids seems to be a promising method, but the application of this method in the practice might require more research under practical conditions. Furthermore, it is important to reveal the mode of action of organic acids in the host digestive tract.
